# Phenotypic, Metabolic, and Functional Characterization of Experimental Models of Foamy Macrophages: Toward Therapeutic Research in Atherosclerosis

**DOI:** 10.3390/ijms251810146

**Published:** 2024-09-21

**Authors:** Amina Sarah Henni Mansour, Mathilde Ragues, Julien Brevier, Coraline Borowczyk, Janaïna Grevelinger, Jeanny Laroche-Traineau, Johan Garaude, Sébastien Marais, Marie-Josée Jacobin-Valat, Edouard Gerbaud, Gisèle Clofent-Sanchez, Florence Ottones

**Affiliations:** 1 CRMSB UMR5536, CNRS DR-15, INSB, 33000 Bordeaux, France; sarah.henni-mansour@rmsb.u-bordeaux.fr (A.S.H.M.); mathilde.ragues@rmsb.u-bordeaux.fr (M.R.); coraline.borowczyk@univ-cotedazur.fr (C.B.); janaina.grevelinger@inrae.fr (J.G.); jlaroche@rmsb.u-bordeaux.fr (J.L.-T.); marie-josee.jacobin-valat@rmsb.u-bordeaux.fr (M.-J.J.-V.); gisele.clofent-sanchez@rmsb.u-bordeaux.fr (G.C.-S.); 2 UMR 7252, XLIM, 87060 Limoges, France; julien.brevier@unilim.fr; 3 MRGM INSERM U1211, 33076 Bordeaux, France; johan.garaude@u-bordeaux.fr; 4 Bordeaux Imaging Center, BIC, UAR 3420, US 4, 33000 Bordeaux, France; sebastien.marais@u-bordeaux.fr; 5 Centre de Recherche Cardio Thoracique, INSERM U 1045, 33000 Bordeaux, France; edouard.gerbaud@chu-bordeaux.fr

**Keywords:** atherosclerosis, foamy macrophages, TPEF autofluorescence, phenotype and cytokine profiling, bioenergetic immunometabolism, cellular oxidative stress, ceroids, antioxidant, immunoregulation, diagnostic and therapeutic strategies

## Abstract

Different types of macrophages (Mφ) are involved in atherogenesis, including inflammatory Mφ and foamy Mφ (FM). Our previous study demonstrated that two-photon excited fluorescence (TPEF) imaging of NADH and FAD autofluorescence (AF) could distinguish experimental models that mimic the different atherosclerotic Mφ types. The present study assessed whether optical differences correlated with phenotypic and functional differences, potentially guiding diagnostic and therapeutic strategies. Phenotypic differences were investigated using three-dimensional principal component analysis and multi-color flow cytometry. Functional analyses focused on cytokine production, metabolic profiles, and cellular oxidative stress, in LDL dose-dependent assays, to understand the origin of AF in the FAD spectrum and assess FM ability to transition toward an immunoregulatory phenotype and function. Phenotypic studies revealed that FM models generated with acetylated LDL (Mac) were closer to immunoregulatory Mφ, while those generated with oxidized LDL (Mox) more closely resembled inflammatory Mφ. The metabolic analysis confirmed that inflammatory Mφ primarily used glycolysis, while immunoregulatory Mφ mainly depended on mitochondrial respiration. FM models employed both pathways; however, FM models generated with high doses of modified LDL showed reduced mitochondrial respiration, particularly Mox FM. Thus, the high AF in the FAD spectrum in Mox was not linked to increased mitochondrial respiration, but correlated with the dose of oxidized LDL, leading to increased production of reactive oxygen species (ROS) and lysosomal ceroid accumulation. High FAD-like AF, ROS, and ceroid accumulation were reduced by incubation with α-tocopherol. The cytokine profiles supported the phenotypic analysis, indicating that Mox FM exhibited greater inflammatory activity than Mac FM, although both could be redirected toward immunoregulatory functions, albeit to different degrees. In conclusion, in the context of immunoregulatory therapies for atherosclerosis, it is crucial to consider FM, given their prevalence in plaques and our results, as potential targets, regardless of their inflammatory status, alongside non-foamy inflammatory Mφ.

## 1. Introduction

Atherosclerosis, a chronic inflammation of medium- and large-size arteries, is characterized by the accumulation of lipids and immune cells in the artery wall. The retention of Low-Density Lipoproteins (LDL) leads to the formation of fat on the inner surface of the artery, which favors LDL oxidation. Consequently, endothelial cell activation facilitates the massive and persistent recruitment of blood monocytes to the intima, where they differentiate into macrophages (Mφ). Blood monocyte-derived Mφ are implicated in all atherogenesis steps [1,2,3,4,5]. These Mφ are highly heterogeneous and plastic, and this variability is particularly evident in atherogenesis [6]. In response to microenvironmental clues, they can be polarized toward an inflammatory phenotype or become foamy Mφ (FM) after the endocytosis of LDL. Plaque progression might be regulated by the delicate balance between atherogenic and immunoregulatory Mφ [7,8,9,10,11]. The accumulation of inflammatory Mφ and dying FM is a hallmark of vulnerable plaque areas. Inflammatory Mφ are clearly pro-atherogenic and have recently been described as predominantly non-foamy [12]. Some studies suggest that inflammatory Mφ might be induced toward immunoregulatory functions as a therapeutic approach to atherosclerosis [13,14]. A crucial issue is whether other major subsets of atherogenic Mφ, specifically FM, might also be targeted by immunoregulatory therapeutic strategies.

The functional status of FM remains highly controversial. Early publications suggested that FM secrete inflammatory cytokines and metalloproteinases that sustain inflammation [15,16]. Conversely, recent results from single-cell technologies have shown that FM possess a non-inflammatory transcriptomic profile [17,18,19,20]. However, single-cell technologies often require cells to be extracted from their microenvironment, which typically results in the loss of FM due to their fragility. Consequently, only a limited number of FM subsets may be analyzed—usually the less foamy ones that are not representative of pathogenic FM. Different FM types exist in atherosclerotic plaques, and their functional status is influenced by various environmental stimuli and the type and quantity of modified LDL trapped within these cells. This was demonstrated by multiplex immunofluorescent and mass spectrometry imaging [21]. Therefore, characterizing the FM panel in plaques is crucial, as FM may exhibit various functional statuses with altered lysosomal function, autophagy, oxidative stress, apoptosis, and necrosis, depending on their lipid load and content [22].

In our previous work, we generated different FM models and analyzed the typical cell surface marker expression patterns associated with inflammatory or immunoregulatory phenotypes using flow cytometry. We found that the different FM models co-express inflammatory and immunoregulatory markers, albeit at low level, and that Two-Photon Excited Fluorescence (TPEF) imaging of NADH and FAD autofluorescence (AF) can distinguish non-foamy inflammatory and immunoregulatory Mφ [23]. This observation was consistent with our initial hypothesis that these two types of Mφ would exhibit distinct optical properties due to the autofluorescence of these cofactors, which are crucial for cellular energy metabolism. Specifically, NADH is primarily produced during glycolysis, while FAD is a key byproduct of mitochondrial oxidative phosphorylation (OXPHOS). It is well established that inflammatory Mφ rely on glycolysis, whereas immunoregulatory Mφ predominantly use OXPHOS pathways to generate the ATP necessary for their functions [24]. However, little was known about the metabolic pathways used by FM. Surprisingly, we observed high AF in both spectra, with particularly elevated FAD-like AF in FM generated with high doses of modified LDL, which was inconsistent with our preliminary metabolism analysis showing low mitochondrial respiration in these macrophages.

In this study, we introduced a broader array of FM models, examining not only the type of LDL (acetylated or oxidized) but also varying doses (low and high). The work presented here has a dual objective. First is to understand why, in TPEF imaging, different FM models exhibit distinct optical properties related to autofluorescence (AF) in the NADH and FAD spectra. In this context, we demonstrated that ceroids are the biological origin of the higher FAD-like AF observed in the Mox model compared to the Mac model. Second is to utilize these results to develop a strategy aimed at restoring atheroprotective functions. In this paper, we focused on immunoregulation and, for the first time, showed that the dysfunctional state of FM can be alleviated by a treatment combining immunoregulatory cytokines and antioxidants.

To determine whether optical differences correlate with phenotypic and functional differences, we first compared the phenotypes (both surface markers expression and natural AF in the NADH and FAD spectra) of different experimental Mφ models: M1 and M2 models, which mimic inflammatory and immunoregulatory Mφ respectively, and two commonly used models to mimic FM, based on Mφ incubated with oxidized LDL (oxLDL; Mox models) or acetylated LDL (acLDL; Mac models). In-depth analysis of multi-color flow cytometry data provided further insights into each experimental FM model. We used the co-expression of cell surface markers to define different subsets (inflammatory, intermediary, immunoregulatory, undefined), the proportions of which suggested that FM models fall differently along the phenotypic continuum from the inflammatory to immunoregulatory extremes (i.e., M1 and M2 models, respectively). Moreover, three-dimensional Principal Component Analysis (3D-PCA) using both flow cytometry and TPEF data clearly discriminated the different experimental Mφ models, particularly distinguishing the Mac model from the Mox model. We then investigated their functional differences by analyzing cytokine secretion profiles and performing metabolic profiling of the different experimental Mφ models. The results confirmed the link between the signal in the NADH spectrum and the use of the glycolysis pathway in all experimental models. Conversely, we could not establish a link between the FAD spectrum signal and the level of FAD produced by mitochondrial respiration. Specifically, FM models generated with a high oxLDL dose displayed higher FAD-like AF and lower mitochondrial respiration compared to those generated with a high dose of acLDL. This suggested a link between the FAD-like AF and the uptake, degradation, and/or storage of modified LDL by FM. As mitochondrial respiration might be influenced by the LDL type and dose, we hypothesized that the mechanism leading to FAD-like AF might involve lipid peroxidation, oxidative stress, and ceroid formation in Mφ. Thus, we measured Reactive Oxygen Species (ROS) production and cellular ceroids in each experimental model. To confirm the role of oxidative stress in producing FAD-like AF, we investigated the effect of the antioxidant alpha-tocopherol. Lastly, we analyzed the functional state of these models by assessing their ability to be polarized toward immunoregulatory Mφ, measuring CD206 surface expression, cytokine secretion profiles, and oxygen consumption. The lower ability of Mox FM, compared to Mac FM, to be polarized highlighted the more dysfunctional state of the Mox models. Overall, this work provides new insights into the functional discrepancies between commonly used FM models and discusses their potential for developing new therapeutic approaches based on the induction of immunoregulatory Mφ in atherosclerosis.

## 2. Results

### 2.1. Phenotypes of the Different Experimental Models of Mφ

- Single-marker flow cytometry analysis and Two-Photon Excited Fluorescence (TPEF) imaging of NADH and FAD suggest differences between the Mox and Mac models

**Figure 1:**
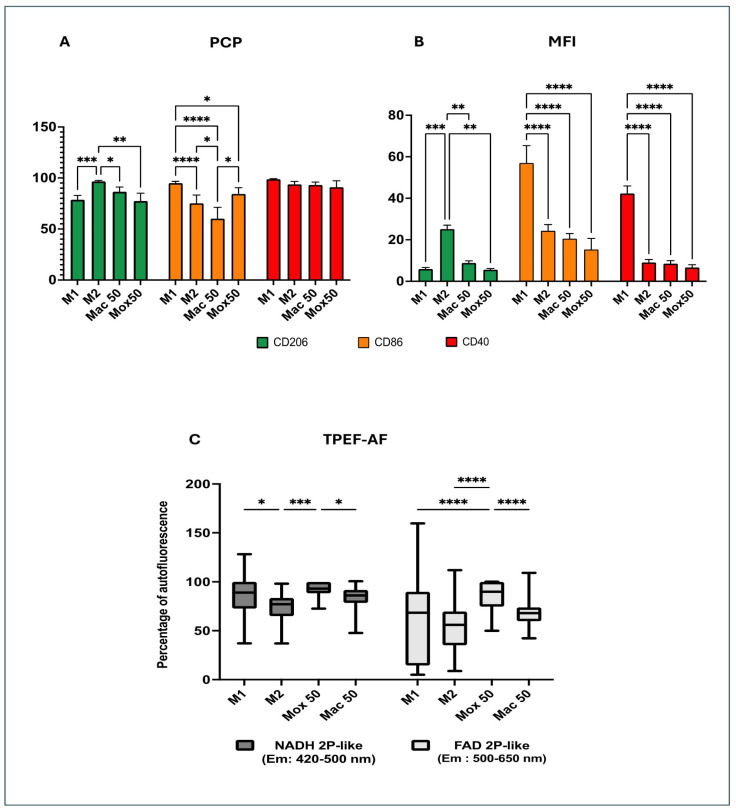
Phenotype comparison of the different experimental models of Mφ: M1, M2, and FM generated with 50 μg/mL of oxLDL (Mox50) or with 50 μg/mL of acLDL (Mac50). (**A**) Mean Fluorescence Intensity (MFI) of CD206, CD86, and CD40 (representing the mean level of expression of each marker at the cell surface); (**B**) Percentage of Cells Positive (PCP) for CD206, CD86, and CD40. (**A**,**B**): data obtained using experimental models derived using blood samples from different donors (n = 5 for Mox, n = 7 for Mac, and n = 13 for M1 and M2) after immunostaining using anti-CD86 and anti-CD206 antibodies. (**C**) FAD and NADH autofluorescence (AF) from TPEF imaging in the NADH spectrum (excitation at 760 nm and detection at 420–500 nm) and FAD spectrum (excitation at 860 nm and detection at 500–650 nm). Normalization was performed for each donor using the maximal value obtained for the experimental model Mox50 as the 100% reference. Data were from n = 8 different donors for all models. * *p*-value < 0.05; ** *p*-value < 0.01; *** *p*-value < 0.001 and **** *p*-value < 0.0001.

In a previous study, we obtained flow cytometry and TPEF data from different Mφ models (M1, M2, and Foamy macrophages (FM)) that mimic the main Mφ types found in atherosclerotic plaques and showed that these experimental models could be distinguished by TPEF imaging [23]. Here, we focused on the comparison of different FM models generated using 50 μg/mL of oxLDL (Mox50) or acLDL (Mac50), respectively. First, we observed that both Mox50 and Mac50 models displayed a low Mean of Fluorescence Intensity (MFI) for the typical immunoregulatory CD206 marker, compared with the M2 model, and for the typical inflammatory CD86 and CD40 markers, compared with the M1 model (Figure 1A). However, the Percentage of Cells Positive (PCP) for CD86 in the Mox50 model was closer to the M1 model. Similarly, the PCP for CD206 in the Mac50 model was closer to the M2 model (Figure 1B). We observed no difference of the PCP CD40 between the different models. The optical features of the Mac50 model were not different from those of M1 and M2 Mφ. Conversely, the Mox50 model presented distinct optical features from all the other models, except for the NADH autofluorescence (AF) level that was similar to that of M1 cells. To note, the AF percentage in the FAD spectrum was higher in the Mox50 model than in M1, in M2, and also Mac50 cells (Figure 1C).

Altogether, these results **highlight the differences between FM models obtained with oxLDL and acLDL. Specifically, Mac50 Mφ are closer to M2 Mφ, and Mox50 Mφ are closer to M1 Mφ.**

- Multicolor flow cytometry analysis of M1- and M2-specific surface marker co-expression suggests that Mox Mφ have more inflammatory features than Mac Mφ.

**Figure 2:**
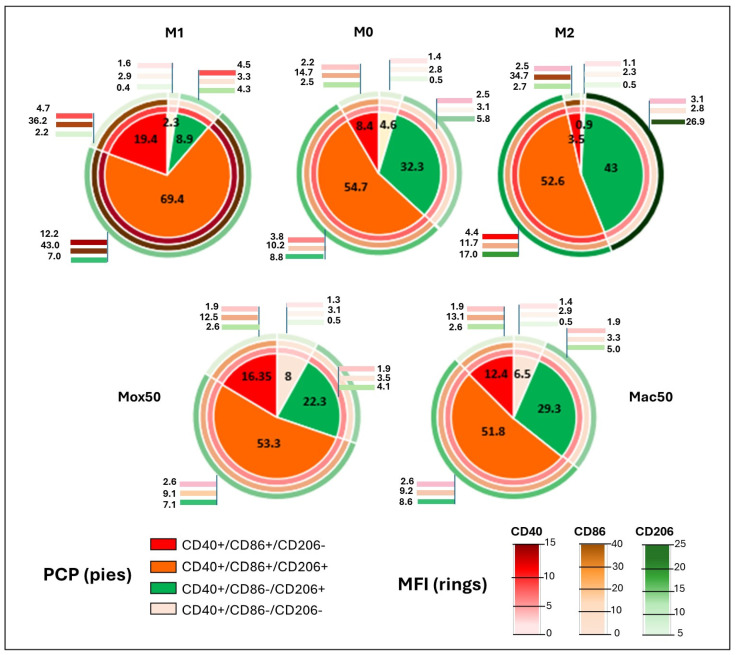
Characterization of the M1, M0, M2, Mox50, and Mac50 models using flow cytometry analysis. Pie charts show the percentage of CD86^+^/CD206^−^, CD86^+^/CD206^+^, CD86^−^/CD206^+^, and CD86^−^/CD206^−^ subsets for each experimental condition. The M0 model, which corresponds to unstimulated control Mφ, shows, as expected, intermediate percentages of the CD86^+^/CD206^−^ and CD86^−^/CD206^+^ subpopulations compared to the two extremes of the continuum, the M1 and M2 models. The outer colored rings indicate the mean fluorescence intensity of CD86 and CD206 using the shown color gradient scale. Data are the mean values of three donors (all cells expressed CD40).

To thoroughly compare the FM model phenotypes, we classified cells in each model in four different sub-populations (see Methods): CD86^−^CD206^−^ (“indeterminate” Mφ), CD86^−^CD206^+^ (“immunoregulatory” Mφ), CD86^+^CD206^+^ (“intermediate” Mφ), and CD86^+^CD206^−^ (“inflammatory” Mφ) (Figure 2). This analysis showed that M1 and M2 polarization resulted in a change of the percentage of each subpopulation and also in the MFI level for CD86 and CD206. M1 polarization was characterized by a reduction of the immunoregulatory (green pie) subpopulations and an increase of the inflammatory (red pie) and intermediate (orange pie) subpopulations, compared with M2. Moreover, in the M1 condition, the expression of CD86 (M1 marker) was increased in the inflammatory and intermediate subsets (MFI of 36.2 and 43) (rings in Figure 2). Conversely, in the M2 condition, the percentage of inflammatory cells was decreased, the percentage of immunoregulatory was increased, and intermediate and immunoregulatory cells strongly expressed CD206 (MFI of 17 and 27) compared with the other experimental conditions.

Comparison of the data for the different FM models showed low expression of M1 and M2 markers without differences between Mox and Mac Mφ, except for the CD206 level that was higher in the Mac50 intermediate subset. Furthermore, the percentage of inflammatory cells was lower and the percentage of the immunoregulatory subset was higher in Mac50 than in Mox50 FM. Thus, this multi-marker analysis confirmed that **Mac50 Mφ are closer to M2 Mφ and Mox50 Mφ are closer to M1 Mφ.**

Altogether, these flow cytometry data suggest that our FM models fit differently in the phenotypic continuum going from the inflammatory to immunoregulatory extremes.

- The 3D-PCA of TPEF and flow cytometry data show clear differences between the Mox and Mac models

**Figure 3:**
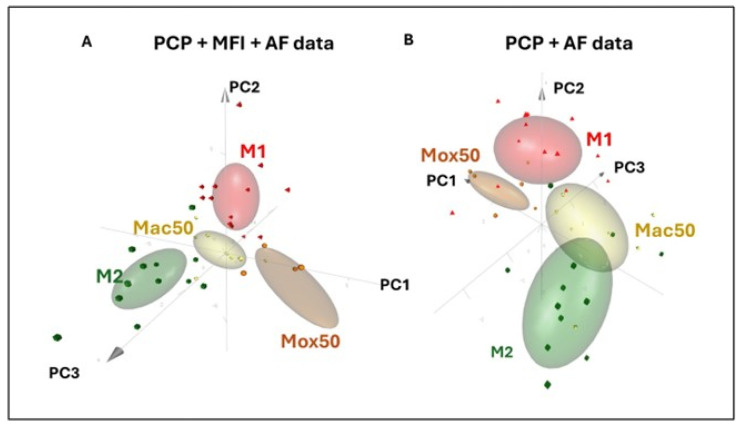
Discrimination of the M1, M2, Mac50, and Mox50 Mφ models using 3D-PCA. (**A**) The 3D-PCA with the PCP, AF, and MFI data and (**B**) 3D-PCA with PCP and AF data. Data obtained from different donors (n = 5 for Mox, n = 7 for Mac and n = 13 for M1 and M2). See Supplemental Figures S1A,B and S2A,B.

To better assess the differences among FM models, we performed 3D-PCA with all collected data (PCP, MFI, and AF values) (Figure 3A) and only with PCP and AF data (Figure 3B). The results for the M1 (red), M2 (green), Mox50 (orange), and Mac50 (yellow) models, represented by confidence ellipses, showed that these populations were clearly separated. The opposite polarization of M1 and M2 Mφ also was clearly visible as well as the spatial separation of the FM models from M1 and M2 cells (Figure 3A). The 3D-PCA performed using only the PCP+AF data highlighted the closer proximity of the Mac50 model with M2 (partial overlap of the green and yellow confidence ellipses) (Figure 3B). The segregation or overlap of the confidence ellipses corresponding to the various experimental conditions can be readily visualized in the 3D video representations generated from captured images, provided in Supplementary Figure S1A,B. The contribution of each individual parameter to the principal components is detailed in Supplementary Figure S2A,B.

The 3D-PCA analysis showed clear differences between FM models: the Mac50 model is closer to M2 Mφ, whereas the Mox50 model is clearly distinct from all the other models.

### 2.2. Functional Comparison of the Experimental Mφ Models

- Cytokine quantification confirms the phenotype differences between Mox and Mac Mφ

**Figure 4:**
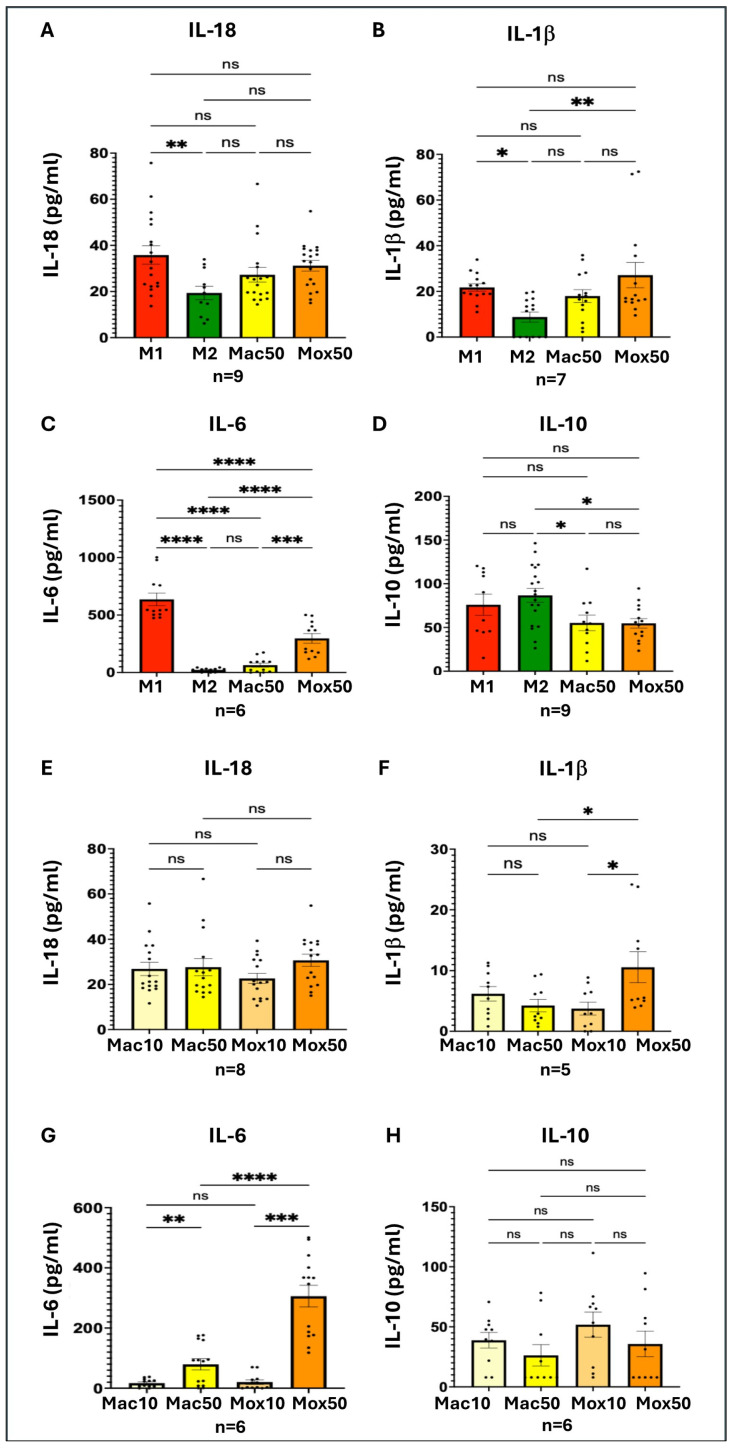
Quantification of the cytokines secreted in the culture supernatant of the different experimental models of Mφ. (**A**) Interleukin-18 (IL-18); (**B**) Interleukin-1β (IL-1 β); (**C**) Interleukin-6 (IL-6); and (**D**) Interleukin-10 (IL-10) in the M1, M2, Mac50, and Mox50 models. Data obtained from different donors (n = 9 for IL-18, n = 7 for IL-1β, n = 6 for IL-6, and n = 9 for IL-10). (**E**) IL-18; (**F**) IL-1β; (**G**) IL-6; and (**H**) IL-10 in the Mac10, Mac50, Mox10, and Mox50 models. Data obtained from different donors (n = 8 for IL-18, n = 5 for IL-1β, n = 6 for IL-6, and n = 6 for IL-10). ns = *p*-value > 0.05; * *p*-value < 0.05; ** *p*-value < 0.01; *** *p*-value < 0.001 and **** *p*-value < 0.0001.

To assess whether the slight phenotypic differences observed between Mac and Mox Mφ translated into functional differences, we assessed the cytokine secretion profiles of the different experimental models. We quantified inflammatory cytokines (IL-18, IL-1β, and IL-6) and IL-10, an immunoregulatory cytokine (Figure 4).

As expected, secretion of inflammatory cytokines was higher in the reference inflammatory M1 model than in the M2 model (Figure 4A–C). The inter-individual variations among donors did not allow appreciating fine variations of cytokine production. IL-18 was similarly produced by Mac50 and Mox50 cells (Figure 4A). Conversely, IL-1β secretion was similar in Mox50 Mφ and the M1 model and was significantly higher than in the M2 model. The wide inter-individual variations of IL-1β production in Mac50 cells did not allow differentiating these Mφ from the two extreme models of the M1/M2 continuum (Figure 4B). Remarkably, IL-6 production was higher in the Mox50 model than in M2 and Mac Mφ (no significant difference between these last models) (Figure 4C). For these experiments, we generated more FM models using low doses (10 μg/mL) of oxLDL (Mox10) and acLDL (Mac10). This allowed showing that the production of inflammatory cytokines was influenced by the oxLDL dose. Indeed, IL-1β and IL-6 production was higher in the Mox50 than in the Mox10 model (Figure 4F,G). On the other hand, IL-10 production was lower in the FM models than in M2 Mφ and not significantly different from M1 Mφ. Altogether, these data suggest that the Mox50 model includes more inflammatory Mφ than the Mac50 model. These results confirmed that **Mox50 Mφ are closer to M1 Mφ and Mac50 Mφ are closer to M2 Mφ.**

- Metabolism studies show clear differences between the Mox and Mac models

As inflammatory cells usually produce ATP via the glycolysis pathway, we first compared the bioenergetic metabolic profile of the Mox50 model and the M0, M1 and M2 models. To this aim, we quantified (1) Oxygen Consumption Rate (OCR), which reflects the mitochondrial respiration flux; (2) ExtraCellular Acidification Rate (ECAR), which reflects the glycolytic flux; and (3) mitochondrial versus non mitochondrial ATP production.

**Figure 5:**
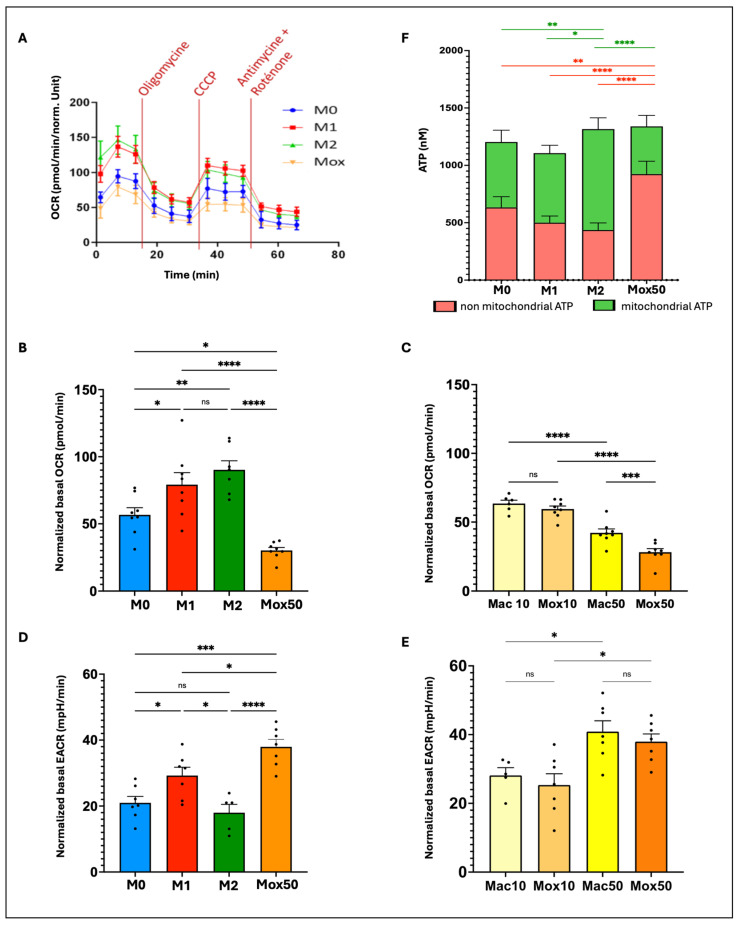
Oxygraphy measurement and ATP production in the different Mφ models. (**A**) Basal Oxygen Consumption Rate (OCR) (basal respiration, ATP-linked respiration, maximal respiratory capacity, and reserve capacity) in M0, M1, M2, and Mox50 cells (representative results; n = 8 different donors). (**B**) Basal OCR in M0, M1, M2, and Mox50 cells (n = 8 donors). (**C**) Basal OCR in Mac10, Mac50, Mox10, and Mox50 cells (n = 8 donors). (**D**) ExtraCellular Acidification Rate (ECAR) indicative of the level of glycolytic flux in M0, M1, M2, and Mox50 cells (n = 7 donors). (**E**) ECAR in Mac10, Mac50, Mox10, and Mox50 cells (n = 7 donors). (**F**) ATP-linked to mitochondrial respiration (light green) versus non-mitochondrial ATP production (light red) in M0, M1, M2, and Mox50 cells (n = 8 donors). Statistical comparisons between experimental conditions are represented by green stars for mitochondrial ATP and red stars for non-mitochondrial ATP. ns = *p*-value > 0.05; * *p*-value < 0.05; ** *p*-value < 0.01; *** *p*-value < 0.001 and **** *p*-value < 0.0001.

Figure 5A shows a representative OCR measurement using the Seahorse technology. OCR values were lower in the Mox50 model compared with the other Mφ models and also with the Mac models (Figure 5B,C), indicating that the high AF observed in the FAD spectrum in Mox50 may not be related to the FAD produced by the mitochondrial respiration. In addition, comparison of the FM models obtained with two different concentrations of modified LDL indicated that the OCR values (and thus mitochondrial respiration) decreased with higher concentrations (Figure 5C). Conversely, ECAR values were higher in the Mox50 than M0 and M2 and even M1 models (Figure 5D). The ECAR values in the Mox50 and Mac50 models were similar and higher than those in Mox10 and Mac10, respectively (Figure 5E). Lastly, quantification of mitochondrial and non-mitochondrial ATP production (Figure 5F) confirmed that the Mox50 model uses mainly the glycolytic pathway.

The finding that Mox50 Mφ display high FAD AF but low mitochondrial respiration suggests that **the signal observed in the FAD spectrum does not rely only on FAD produced by the OXPHOS pathway**, particularly in the FM models generated using the higher oxLDL concentration.

### 2.3. Determination of the Biological Origin of the High FAD-like AF in Mox50 Mφ

- The FAD AF is influenced by the LDL dose and is higher in Mox than in Mac Mφ

**Figure 6:**
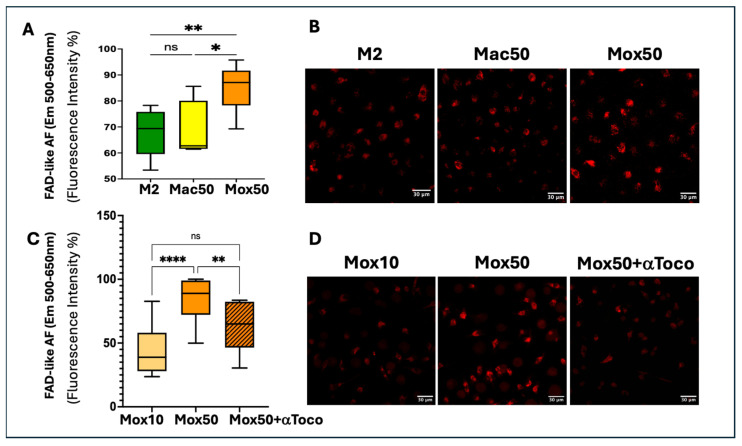
The AF in the FAD spectrum is linked to LDL handling and oxidative stress. (**A**) Quantification of FAD-like AF (λ_Em_= 500–650 nm) in M2, Mac50, and Mox50 cells (n = 8). (**B**) Representative TPEF images of one donor (n = 8) in the M2, Mac50, and Mox50 conditions. (**C**) Quantification of AF emission in the FAD spectrum (Em 500–650 nm) in the Mox10, Mox50, and Mox50 + α-tocopherol conditions (n = 4). (**D**) Representative TPEF images of one donor (n = 3), in the Mox10, Mox50, and Mox50 + α-tocopherol conditions. ns = *p*-value > 0.05; * *p*-value < 0.05; ** *p*-value < 0.01; **** *p*-value < 0.0001.

The AF emitted in the FAD spectrum measured by TPEF was significantly higher in the Mox50 than in the Mac50 model and also the M2 model (Figure 6A,B), unlike what was observed for the OCR values (Figure 4B,C). This suggested that the AF observed in the FAD spectrum was due to another molecule. The level of this FAD-like AF was also significantly higher in Mox50 Mφ than in Mox10 Mφ and decreased when α-tocopherol (an antioxidant) was simultaneously used with oxLDL (Figure 6C,D).

As the AF signal intensity was proportional to the oxLDL concentration used to generate the Mox models and was significantly decreased by α-tocopherol, we hypothesized that **the AF signal in the spectrum of FAD might be linked to oxLDL uptake and the subsequent cellular oxidative stress.** Therefore, we quantified ROS production in the Mox and Mac models (with and without α-tocopherol) and compared the lysosomal accumulation of ceroids in both models.

- The levels of ROS and cellular ceroids are higher in Mox than in Mac Mφ

**Figure 7:**
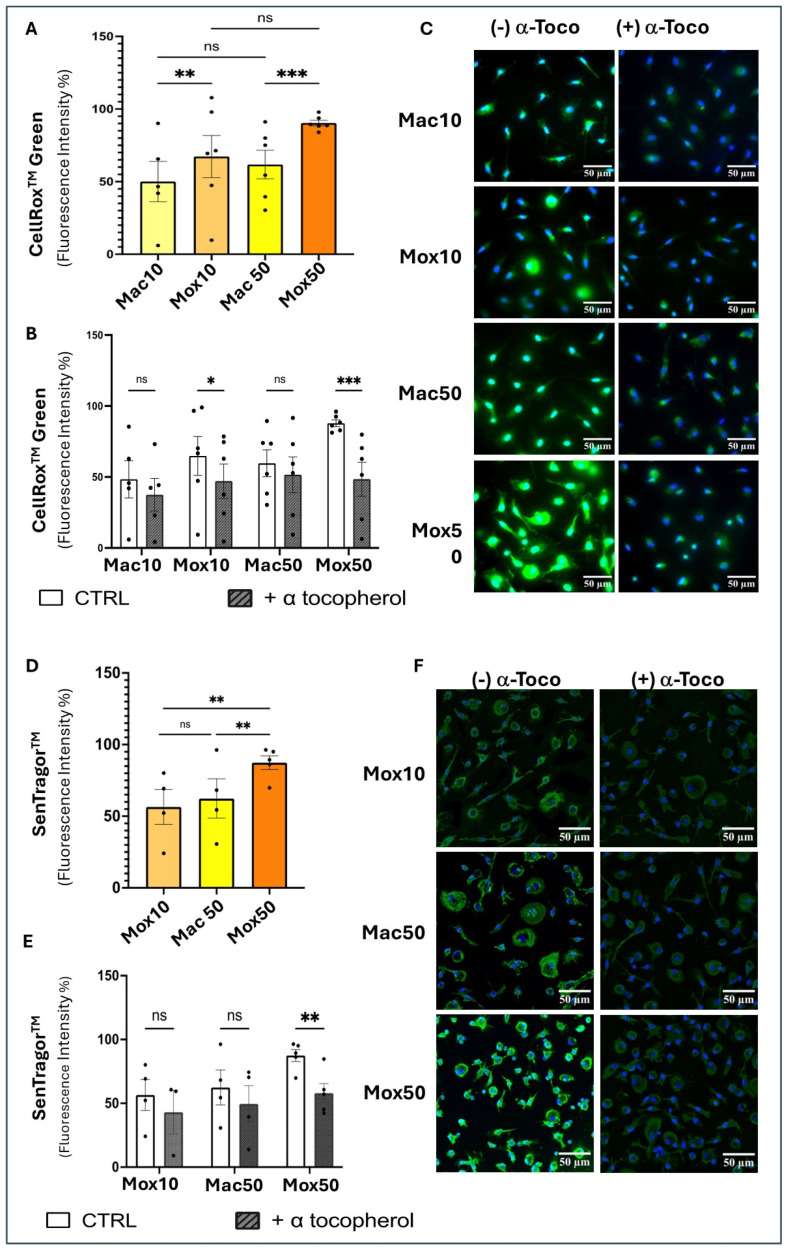
Detection of cellular reactive oxygen species (ROS) and ceroids. (**A**) Quantification of ROS level in the different experimental models of FM (Mac10, Mox10, Mac50, Mox50) after labeling with CellROX (n = 6 donors) (expressed as percentage of the maximal fluorescence intensity observed in the Mox50 models for each donor). (**B**) Quantification of ROS in the different models (Mox10, Mox50, Mac10, Mac50) obtained in the presence or absence of the antioxidant α-tocopherol (n = 6 donors). (**C**) Fluorescence microscopy images of the different models (Mox10, Mox50, Mac10, Mac50) after CellROX labeling (n = 6 donors). (**D**) Quantification of the SenTraGor^TM®^ fluorescence intensity in the different FM models (Mox10, Mac50, Mox50) (n = 6 donors). (**E**) Quantification of SenTraGor^TM®^ fluorescence intensity in the presence or absence of the antioxidant α-tocopherol (n = 6 donors). (**F**) Confocal microscopy images of the different models generated in the presence or absence of α-tocopherol and labeled with SenTraGor^TM®^ (n = 6 donors). ns = *p*-value > 0.05; * *p*-value < 0.05; ** *p*-value < 0.01; *** *p*-value < 0.001.

To investigate the implication of cellular oxidative stress, we evaluated ROS production in the different FM models in the presence or absence of α-tocopherol. ROS production was higher in the Mox than in the Mac models (Figure 7A) and was decreased by co-incubation with α-tocopherol (Figure 7B,C). As oxidative stress may result in lipid peroxidation and lysosomal accumulation of ceroids, we quantified ceroids in the different models using a derivative of Sudan Black, SenTraGor^TM®^, which binds specifically to cellular ceroids. As observed for the FAD-like AF, the level of ceroids was higher in Mox50 than in Mac50 and Mox10 cells (Figure 7D) and markedly decreased in the presence of α-tocopherol (all models) (Figure 7E,F).

**Figure 8:**
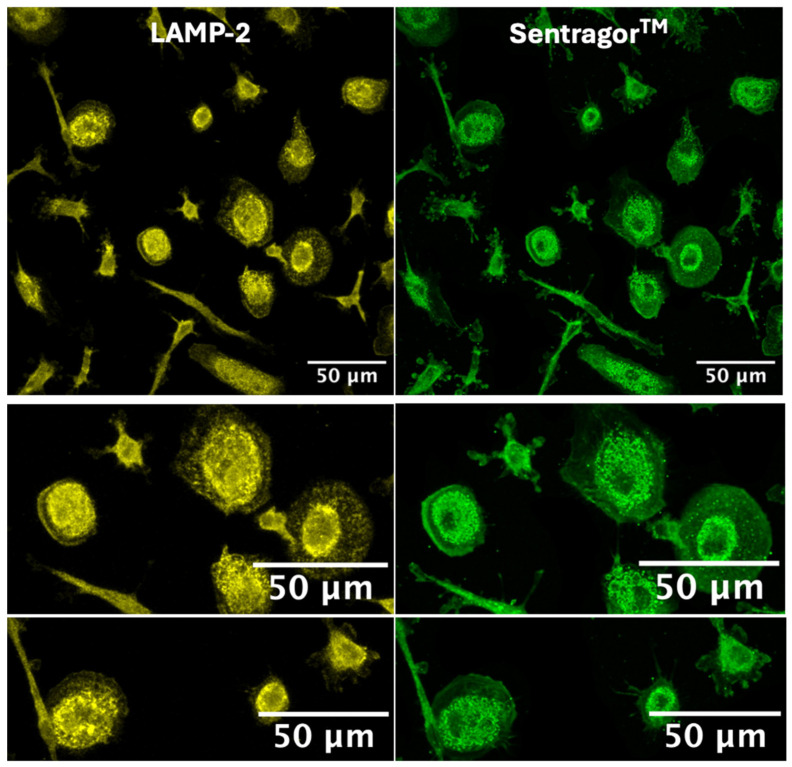
Colocalization of ceroids and LAMP-2 (lysosomal marker) in the Mox50 model (representative images obtained from one donor, n = 3, with a Leica TCS SP5 microscope).

Altogether, in Mox50, the AF signal (λ_Em_= 500–650 nm) and ceroid presence decreased when FM were generated in the presence of the antioxidant α-tocopherol that limited ROS production in these cells. These results support the hypothesis that ceroids are the biological origin of the FAD-like AF signal in our FM models. This was further supported by the colocalization of SenTraGor^TM®^-positive ceroids with LAMP2, a lysosome marker (Figure 8).

All these findings support the conclusion that Mφ in the Mox model exhibit a more inflammatory profile compared to those in the Mac model. Additionally, these Mφ are in a more dysfunctional state, as evidenced by reduced mitochondrial respiration, increased oxidative stress, and ceroid accumulation, indicating lysosomal dysfunction.

### 2.4. Evaluation of the Effects of α-Tocopherol and/or Type-2 Cytokine Cocktail on the Phenotypes and Functions of These FM Models

- The antioxidant α-tocopherol influences the energetic metabolism in both FM models

**Figure 9:**
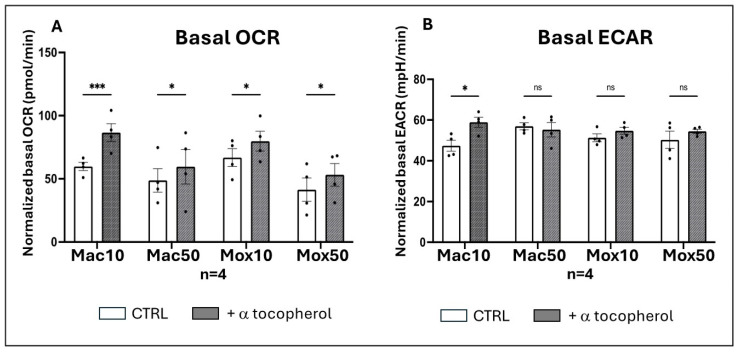
Oxygraphy measurement in the different FM models generated in the absence or in the presence of α-tocopherol. (**A**) Basal Oxygen Consumption Rate (OCR) indicative of the level of basal mitochondrial respiration in Mac10, Mac50, Mox10, and Mox50 Mφ incubated or not with α-tocopherol (n = 4 donors), (**B**) Basal ExtraCellular Acidification Rate (ECAR) indicative of the level of glycolytic flux in Mac10, Mac50, Mox10, and Mox50 Mφ incubated or not with α-tocopherol (n = 4 donors). ns = *p*-value > 0.05; * *p*-value < 0.05; *** *p*-value < 0.001.

To determine whether decreasing the cellular oxidative stress could modify the energetic pathways in both FM models, we generated FM models in the presence of α-tocopherol and used oxygraphy. The basal OCR values were increased in both Mac and Mox models generated by co-incubation with modified LDL and α-tocopherol compared with models generated by incubation with modified LDL alone. Notably, the effect was more pronounced when acLDL were used at a low dose, suggesting that cell dysfunction is more severe (and therefore more difficult to restore) with oxLDL (regardless of the dose) and with a high dose of acLDL (Figure 9A). The antioxidant treatment had no effect on the level of glycolysis in the cells, except for a slight increase of glycolysis in the Mac10 cells (Figure 9B).

The restoration of Mφ mitochondrial respiration using α-tocopherol suggests that the diminution of ceroids in atherosclerotic plaques observed in vivo after α-tocopherol treatment [25] might be partly linked to the metabolic shift of cells toward the energetic pathway preferentially used by immunoregulatory M2.

- A type-2 cytokine cocktail induces the M2 phenotype in Mac and Mox Mφ.

**Figure 10:**
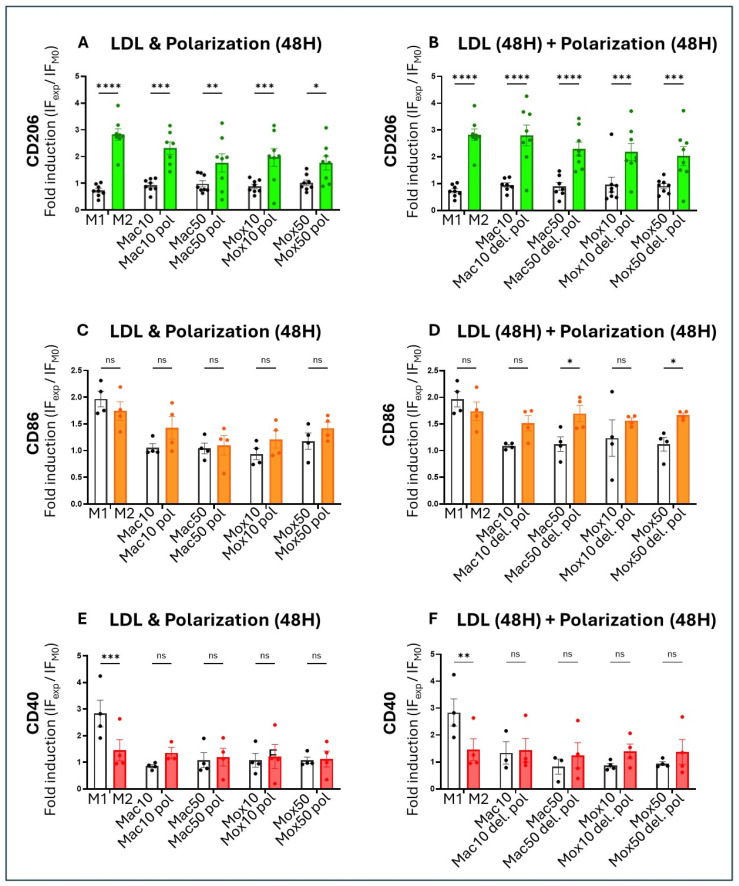
Fold induction of the different surface markers (MFI of the marker in the experimental condition divided by its MFI in the control condition, M0 = unstimulated Mφ) for the ‘Polarization’ protocol (stimulation with the cytokine cocktail added simultaneously with oxLDL/acLDL for 48 h): (**A**) CD206 (n = 8 donors); (**C**) CD86 (n = 4 donors); and (**E**) CD40 (n = 4 donors), or for the ‘Delayed Polarization’ protocol (treatment with oxLDL/acLDL for 48 h, followed by the cytokine cocktail for 48 h after changing the medium): (**B**) CD206 (n = 8 donors); (**D**) CD86 (n = 4 donors); and (**F**) CD40 (n = 4 donors). Here ‘Mac/Mox’ models were named ‘Mac/Mox pol’ (for polarization) when the cytokine cocktail was added during LDL incubation or ‘Mac/Mox del. pol’ (for delayed polarization) when the cytokine cocktail was added after the incubation with LDL, once the cells had already become FM. ns = *p*-value > 0.05; * *p*-value < 0.05; ** *p*-value < 0.01; *** *p*-value < 0.001 and **** *p*-value < 0.0001.

To address the capacity of FM to be polarized toward the M2 phenotype, we generated Mac and Mox models by co-incubating cells with a cocktail of type-2 cytokines (IL-4 and IL-13) and LDL for 48 h (“Polarization”) or added the cocktail for 48 h after the 48 h incubation with LDL (“Delayed polarization”). In this case, the medium was changed before incubation with the cytokine cocktail. Then, we evaluated CD206, CD86, and CD40 cell surface expression (Figure 10). We calculated the fold induction of each marker in a specific experimental condition compared to the control condition (unstimulated Mφ). We observed a significant increase in CD206 cell surface expression in all FM models incubated with the cytokine cocktail during LDL uptake (Figure 10A) and afterward (Figure 10B). This suggests that both Mac and Mox models might switch to the M2 phenotype. The increase was lower with the higher doses of modified LDL (Figure 10A). In addition, when stimulation with the cytokines was performed after incubation with LDL (thus, when cells had become FM), CD206 expression increase was significantly lower in the Mox models than in the Mac models (Figure 10B). CD86 expression did not vary significantly after co-incubation with LDL and the cytokine cocktail (Figure 10C), but slightly increased in all FM models when polarization was induced after LDL uptake by Mφ (Figure 10D). CD40 expression was not affected by both polarization protocols (Figure 10E,F).

**Figure 11:**
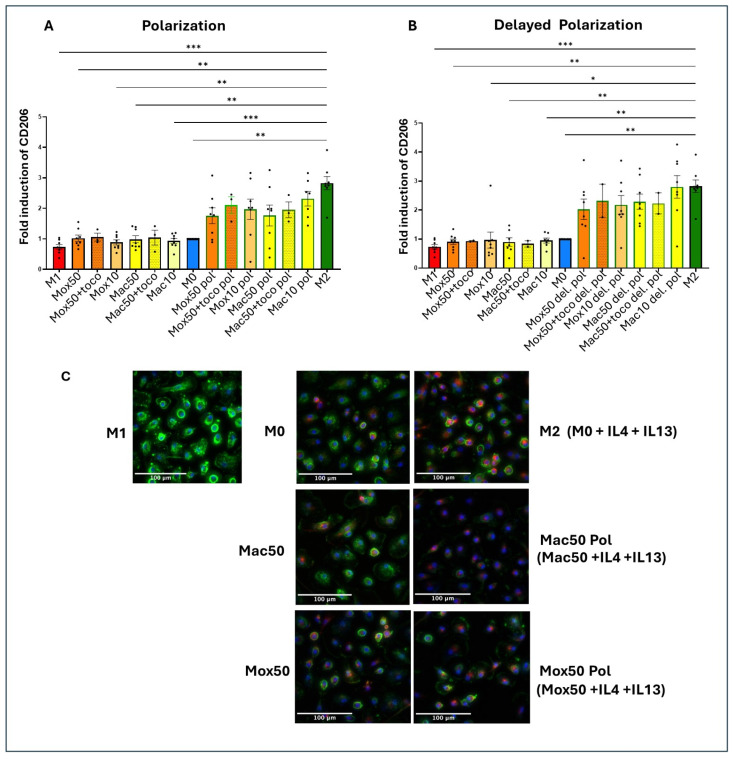
Degree of polarization (represented by the fold induction of CD206) according to the experimental conditions: (**A**) for the ‘Polarization’ protocol, where the cytokine cocktail, alone or with α-tocopherol, is added simultaneously with oxLDL/acLDL for 48 h, and (**B**) for the ‘Delayed Polarization’ protocol, when oxLDL/acLDL alone or in combination with α-tocopherol was added for 48 h, followed (after changing the medium) by the cytokine cocktail for 48 h. The Mac/Mox models were named ‘Mac/Mox pol’ (for polarization) when the cytokine cocktail (to induce polarization) was added during LDL incubation, or ‘Mac/Mox del. pol’ (for delayed polarization) when the cytokine cocktail was added after incubation with LDL, once the cells had already become FM. Data for the histograms were obtained by automated quantification of the CD40 and CD206 fluorescence signals in the imaged cells (representative images obtained from one donor, n = 4). (**C**) Images of Mφ obtained after a 48 h incubation with ac/oxLDL alone, or in combination with the cytokine cocktail. * *p*-value < 0.05; ** *p*-value < 0.01; *** *p*-value < 0.001.

In either case (polarization or delayed polarization), the different experimental conditions can be classified along the inflammatory-to-immunoregulatory continuum (or the “M1-to-M2 polarization continuum”) depending on the CD206 fold induction (Figure 11). This classification clearly showed that all the cells treated with cytokines with or without α-tocopherol expressed CD206 at the same level than the reference M2 condition. Addition of α-tocopherol (always in the first 48 h of incubation with LDL) did not induce any additional effect compared with the cocktail of cytokines alone (added at the same time, Figure 11A, or after the LDL, Figure 11B).

To illustrate this experiment, some representative images are shown in Figure 11C. M1 cells strongly expressed CD40 (in green) and weakly expressed CD206 (in red). Conversely, M2 cells strongly expressed CD206 and weakly expressed CD40. Regarding FM models, CD206 expression was also clearly increased in both Mac and Mox models by using the cytokine treatment (Figure 11C).

Altogether, these findings demonstrated that Mac and Mox Mφ can be polarized toward an M2-like phenotype.

- The type-2 cocktail might induce immunoregulatory functions

**Figure 12:**
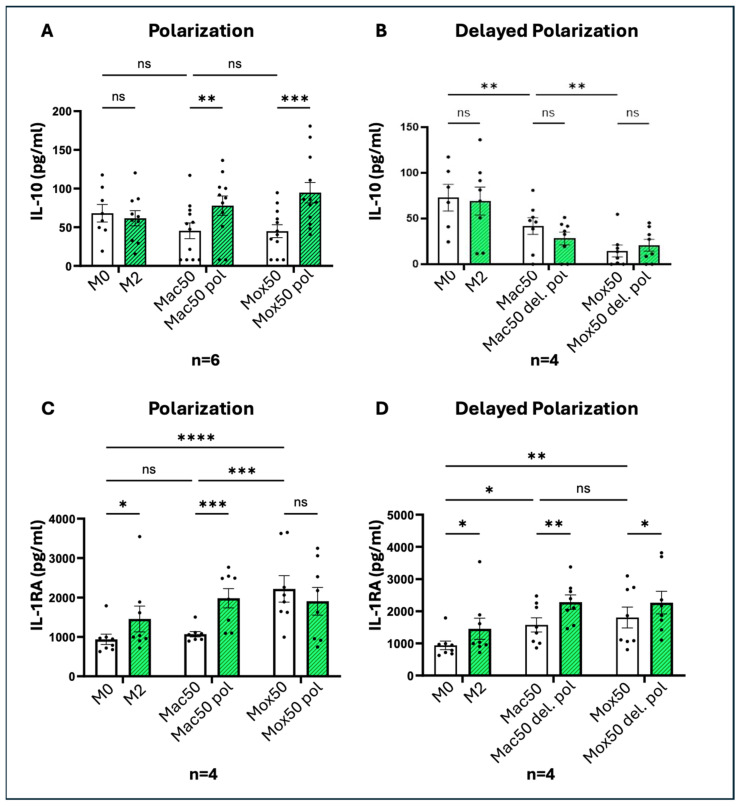
Quantification of the production of type-II cytokines after treatment with the immunoregulatory cytokine cocktail. IL-10 production in the Mac50 and Mox50 models: (**A**) stimulated with the cytokine cocktail added simultaneously with oxLDL/acLDL (polarization) (n = 6 donors), or (**B**) stimulated with the cytokine cocktail added after the incubation with modified LDL (delayed polarization) (n = 4 donors); IL1-RA production in the Mac50 and Mox50 models: (**C**) stimulated with the cytokine cocktail added simultaneously with oxLDL/acLDL, or (**D**) stimulated with the cytokine cocktail added after incubation with modified LDL (delayed polarization) (n = 4 donors). ns = *p*-value > 0.05; * *p*-value < 0.05; ** *p*-value < 0.01; *** *p*-value < 0.001 and **** *p*-value < 0.0001.

To determine whether the different FM models can be polarized toward M2-like functions, we quantified the cytokines secreted by Mox50 and Mac50 Mφ following stimulation, as previously described. The secretion of IL-10 by both Mac50 and Mox50 cells was significantly increased compared to untreated cells only after co-incubation with LDL and the cytokine cocktail (Figure 12A), but not after stimulation of cells that were already differentiated into FM (Figure 12B).

We also measured the secretion of IL-1RA, another immunoregulatory cytokine. Basal production of IL-1RA was notably high in Mox50 cells, significantly higher than in Mac50 and M2 cells. Consequently, we only observed an increase in IL-1RA secretion in Mac50 cells after co-incubation with LDL and the type-II cytokine cocktail (Figure 12C). In contrast, when the cytokine cocktail was added after LDL, we saw a significant increase in IL-1RA production in both models, though the increase was less pronounced in Mox50 Mφ (Figure 12D).

These results showed that both models can be polarized toward immunoregulatory functions, with the lower reactivity of Mox FM reflecting their more dysfunctional state.

- Incubation with the type-2 cytokine cocktail and α-tocopherol reduces drastically IL-6 production by Mox50 cells

**Figure 13:**
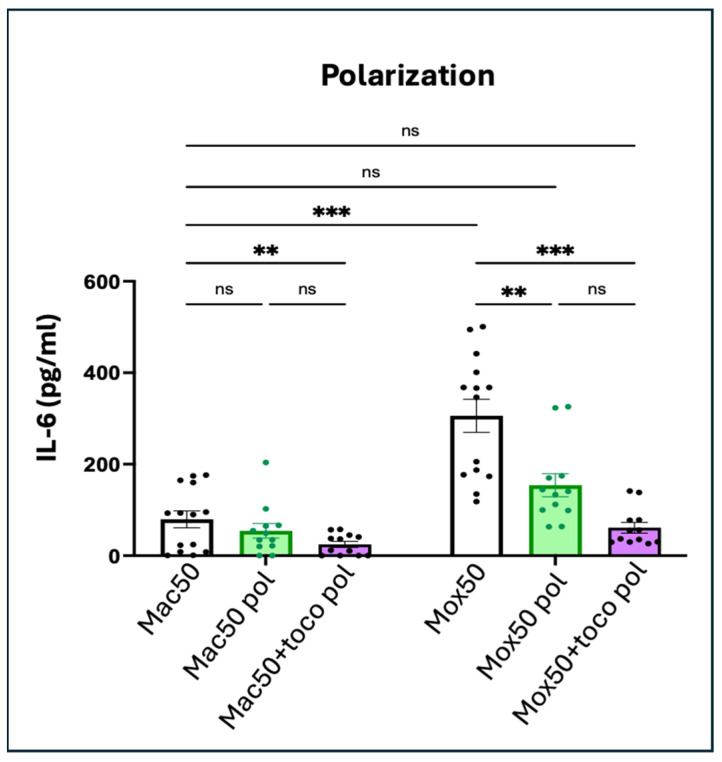
Quantification of IL-6 production in the Mac50 and Mox50 models stimulated with the cytokine cocktail, added simultaneously with oxLDL/acLDL, with or without α-tocopherol (n = 6 donors). Cells were incubated with acLDL/oxLDL (Mac50/Mox50), with acLDL/oxLDL + IL-4 + IL-13 (Mac50/Mox50 pol) or with acLDL/oxLDL + α-tocopherol + IL-4 + IL-13 (Mac50/Mox50 + toco pol). ns = *p*-value > 0.05; ** *p*-value < 0.01; *** *p*-value < 0.001.

Mox50 Mφ generated in the presence of oxLDL combined with IL-4 and IL-13 exhibited significantly reduced IL-6 production compared to Mφ treated with oxLDL alone (Figure 13). This effect was further enhanced when α-tocopherol was also added, resulting in IL-6 levels comparable to those seen in Mac50 Mφ (whose IL-6 production was similar to the M2 model shown in Figure 4C). These findings suggested that **the cytokine cocktail effectively reduces the inflammatory profile of Mox50** Mφ.

## 3. Discussion

Recent advances in cytometry by time of flight and single-cell RNA sequencing have revealed the presence of numerous distinct Mφ subsets within plaques. This diversity can be attributed to the high plasticity of Mφ, fostered by microenvironmental cues. These subsets can be broadly classified into pro-atherogenic Mφ (including non-foamy inflammatory Mφ and dysfunctional Mφ) and anti-atherogenic Mφ (including non-foamy immunoregulatory Mφ). Foamy macrophages (FM) have long been considered as a key feature of atherosclerotic plaques, being abundant in vulnerable plaques during disease progression.

Traditionally, FM were categorized as inflammatory M1-like cells. However, Chinetti-Gbaguidi et al. [26] proposed the existence of various FM types, and Medbury et al. observed that FM can display heterogeneity and co-express both M1 and M2 markers, such as CD86 and CD206 [27]. More recent single-cell analyses identified FM as M2-like immunoregulatory cells [17,18,19,20]. Despite these insights, single-cell analyses may miss fragile FM subpopulations due to the limitations of cell extraction methods. Goossens et al. [21] utilized ex vivo multispectral imaging to map FM phenotypes in tissue samples, identifying several FM subsets, including one correlated with the TREM2^hi^ FM subset identified by single-cell RNA sequencing [28]. Nevertheless, the specific roles of each FM subset remain unclear. FM likely represent a complex population with diverse functions, including some involved in lipid clearance or plaque progression.

In our previous study, we discriminated inflammatory and immunoregulatory Mφ using TPEF imaging of NADH and FAD, which are produced in the glycolysis and OXPHOS pathways, respectively. At that time, the energetic metabolism pathway associated with FM was unknown. Using FM models made with acLDL and oxLDL (Mac and Mox models), we observed unexpectedly high TPEF imaging signals in the FAD spectrum, particularly in Mox FM. Additionally, the expression levels of CD86 and CD206 in Mox were lower compared to those in the M1 and M2 models, respectively.

Our multi-marker analysis revealed key differences between the Mac and Mox FM models. Mox FM had a higher percentage of the CD86^+^/CD206^−^ subset and a lower percentage of the CD86^−^/CD206^+^ subset compared to Mac FM. The NADH AF signal in Mox FM was similar to that in M1 Mφ, associated with a very high AF signal in the FAD spectrum. The 3D-PCA of flow cytometry and TPEF data suggested that Mac FM are closer to M2 cells, while the Mox model is distinct from all the other experimental models. Overall, the Mac and Mox FM models differ in surface marker expression and endogenous natural AF, allowing us to distinguish various Mφ types using 3D-PCA.

We then investigated the functional and energetic metabolic profiles of the Mac and Mox models using low (10 μg/mL: Mac10, Mox10) or high doses (50 μg/mL: Mac50, Mox50) of modified LDL.

Cytokine secretion comparison has focused on three inflammatory cytokines and two immunoregulatory cytokines. The inflammatory cytokines, IL-1β, IL-6, and IL-18, are recognized as key drivers of the athero-inflammatory process [29,30,31], two of which, IL-1β and IL-6, are already the targets of therapeutic strategies [32,33,34]. We also chose to quantify the immunoregulatory cytokines, IL-10, which has shown strong potential as a therapeutic target [35,36,37] and IL-1RA, an antagonist of IL-1 even if its therapeutic application in cardiovascular diseases remains unproven [38].

Cytokine quantification in Mox and Mac models revealed that both FM models produced high levels of the inflammatory cytokines IL-18 and IL-1β, similar to M1. Compared to Mac cells, Mox50 cells produced significantly more IL-6 but less IL-10. The inflammatory state of Mox models was dose-dependent, with higher IL-1β and IL-6 production in Mox50 than Mox10. This cytokine profiling confirmed that the Mox50 model contains more inflammatory cells than the Mac50 model.

These findings align with our metabolic analyses. Inflammatory Mφ primarily rely on glycolysis, whereas immunoregulatory Mφ depend more on mitochondrial respiration. The Mox50 model exhibited higher glycolysis and lower mitochondrial respiration than the M1 model, indicating a metabolic similarity to inflammatory Mφ. The Mac50 model also relied on glycolysis. These results suggest that the AF observed in the NADH spectrum in both Mox50 and Mac50 models is likely due to NADH produced during glycolysis. Conversely, the Mox50 model showed reduced mitochondrial respiration and higher FAD-like AF compared with the Mac50 model, ruling out the possibility that the strong AF in the FAD spectrum originated from mitochondrial FAD production. Furthermore, the FAD-like AF signal was higher and mitochondrial respiration was lower in the Mox50 model compared with the Mox10 model. The increased FAD-like AF and reduced mitochondrial respiration in Mox50 cells led us to hypothesize that this AF is associated with a more pronounced dysfunctional state in Mox50 Mφ.

To address this question, we compared ROS production and lysosomal ceroid accumulation across the different FM models. Ceroids are aggregates of oxidized proteins and lipids with natural AF that overlaps with the FAD spectrum. Mox cells are associated with dysfunctional lysosomes, leading to ceroid deposition and impaired cholesterol efflux [39,40], mitophagy, and autophagy, along with inflammation, senescence. and apoptosis [22]. A recent study from Albaghdadi M.S. et al. [25] highlighted ceroid as a key source of near-infrared autofluorescence (NIRAF) in human atherosclerosis and demonstrated that oxLDL induces (NIRAF), oxidative stress, and production of lipid peroxidation products in Mφ, which were decreased by a treatment with antioxidants (α-tocopherol and N-acetylcysteine (NAC). In accordance with their study, our results showed that ROS production and ceroid accumulation were higher in Mox50 cells. Antioxidant α-tocopherol reduced FAD-like AF, ROS production, and ceroid accumulation in the Mox50 model, suggesting a connection between this AF and oxLDL handling. Additionally, the comparison of different lipid types and doses showed that they may induce Mφ with varying functional profiles. The Mox50 model may represent FM with more severe dysfunction due to impaired oxLDL handling, resulting in higher cellular stress, ROS production, and lysosomal accumulation of ceroids. This underscores the importance of developing different experimental models to mimic different plaque FM subsets. While these models may imperfectly replicate plaque Mφ, they could represent Mφ with varying lipid loads and dysfunction levels, which may be used to explore therapeutic approaches aimed at restoring atheroprotective functions, such as blocking inflammation, promoting cholesterol efflux, or inducing immunoregulation.

The effects of acLDL and oxLDL on cholesterol metabolism in Mφ have been extensively studied by other groups. OxLDL induces more severe lysosomal damage compared to acLDL. While acLDL is efficiently hydrolyzed and allows cholesterol efflux, oxLDL is less effectively degraded, leading to cholesterol accumulation, crystal formation, and altered lysosomal pH. This disruption impairs lysosomal hydrolase activity, causing significant lysosomal dysfunction marked by increased ceroid formation [40,41,42,43,44,45]. These findings are consistent with our AF and SenTraGor^®^ imaging results, indicating that oxLDL is associated with greater functional impairment of lysosomes than acLDL. Although cholesterol efflux is a well-established therapeutic approach to ameliorating the condition of FM, our study deliberately focused on an alternative strategy involving the induction of immunoregulation in FM.

We thus assessed whether the different FM models (Mox10, Mac10, Mox50, and Mac50 cells) could be polarized toward an immunoregulatory profile using immunoregulatory cytokines (IL-4 and IL-13) according to two protocols: (1) co-incubation of Mφ with modified LDL and the cytokine cocktail (“polarization”) to mimic Mφ that have recently infiltrated the intima and are being exposed to both oxLDL and therapeutic treatment, and (2) initial incubation with modified LDL, followed by cytokine stimulation (“Delayed Polarization”) to mimic the effect of the treatment on Mφ that have already taken up modified LDL and are potentially dysfunctional. In some experiments, α-tocopherol was added to the modified LDL to test whether this antioxidant could improve the functional state of FM and enhance their response to the immunoregulatory treatment.

CD206 expression was significantly increased in all FM models incubated with the cytokine cocktail, both during and after LDL uptake. IL-10 secretion increased significantly when cytokines were added during LDL incubation, but not after FM formation, suggesting that established FM may be less amenable to M2 polarization. The high IL-10 production in M1 Mφ (and also in unstimulated M0 Mφ) was unexpected, although it has been previously observed in a similar M1 model [45].Therefore, we measured the production of IL-1RA, another well-known immunoregulatory cytokine. IL-1RA production increased in the Mac50 model when the cytokine cocktail was added early, but not in the Mox50 model. This lack of increase in the Mox50 model may be due to already high levels of IL-1RA present in untreated Mox50 cells. Sequential incubation (LDL first, followed by cytokine stimulation) increased IL-1RA in both models, but to a lesser extent in Mox50, suggesting greater dysfunction and reduced potential for immunoregulatory polarization. IL-6 production in Mox50 cells decreased with cytokine treatment, and α-tocopherol addition further reduced it to levels similar to Mac50 and M2 conditions, indicating that α-tocopherol may alleviate inflammation and dysfunction caused by high oxLDL concentration. Further comparative studies with antioxidants with superior efficacity, such as probucol, could provide insights into more effective treatments [46].

In summary, our study provides insights into the functional profiles of FM models and their potential use in screening drugs with anti-inflammatory or immunoregulatory effects. Both acLDL- and oxLDL-induced FM models could serve as useful tools for evaluating drugs, as they may reflect FM at an early or late stage, respectively. The choice of the model should align with the conceptual framework and experimental objectives. The Mac model could be more suitable for evaluating whether drugs or biologics (e.g., therapeutic antibodies) can repolarize early FM to an immunoregulatory phenotype. The Mox50 model, representing a more advanced stage of FM, might be used to assess therapeutics aimed at restoring cholesterol efflux and reversing LDL handling dysfunction.

## 4. Materials and Methods

### 4.1. Monocyte Isolation, Differentiation, and Stimulation to Generate the Experimental Models of Mφ

Monocytes were negatively selected (PAN BioTech kit, Miltenyi Biotec, Paris, France) from peripheral blood mononuclear cells (PBMC) isolated using Ficoll-Paque Premium 1.073 density gradient (GE Healthcare, Strasbourg, France) from blood samples of healthy donors (Etablissement Français du Sang (EFS) of Bordeaux, Bordeaux, France). A quantity of 3–5 × 10^5^ purified monocytes/mL were seeded on round glass coverslips (for cell imaging) in 12-well plates and differentiated into monocyte-derived macrophages by 5–6-day culture in complete medium (RPMI-1640 medium (Dutscher, Bernolsheim, France) with 10% heat-inactivated AB human serum (Dutscher), 100 U/mL penicillin (Dutscher), and 100 µg/mL streptomycin (Dutscher)) containing 20 ng/mL recombinant human (rh) M-CSF (Miltenyi Biotec). Half of the medium was replaced with fresh medium once during culture. After the differentiation period, cultures were rested for 24 h (complete medium without M-CSF) before a 48 h incubation with the following: (1) 20 ng/mL of rhIFN-γ (Miltenyi Biotec) plus 100 pg/mL of lipopolysaccharide (LPS) (cell culture-suitable, γ-irradiated, BioXtra LPS, Sigma Aldrich) for the classically activated M1 model; (2) 20 ng/mL of rhIL-4 and rhIL-13 (Miltenyi Biotec) for the alternatively activated M2 model; (3) 10 and 50 µg/mL of human oxLDL (Thermo Fischer Scientific France, Illkirch-Graffenstaden, France) for the Mox10 and Mox50 models; (4) 10 and 50 µg/mL of human acLDL (Thermo Fischer Invitrogen) for the Mac10 and Mac50 models. In some experiments, α-tocopherol (Sigma Aldrich, St-Quentin-Fallavier, France) was added in combination with LDL. In some experiments (polarization assays), 20ng/mL of both IL-4 and IL-13, with or without 1 mM α-tocopherol, were combined with modified LDL for a 48 h stimulation before assessing CD206 surface marker expression or cytokine production.

### 4.2. TPEF Data Acquisition

Three-dimensional autofluorescence (AF) images were acquired with a Leica TCS SP5 system (Leica Microsystems, Mannheim, Germany) using the two-photon excitation fluorescence (TPEF) modality and analyzed as described previously [23,47]. Briefly, imaging in the NADH fluorescence spectral band was performed at 760 nm excitation and 420–500 nm detection. Imaging in the FAD fluorescence spectral band was performed at 860 nm excitation and 500–650 nm detection. Taking advantage of intrinsic optical sectioning of two-photon excitation, the AF volume densities of individual cells were extracted in both spectral bands.

### 4.3. Combined Analysis of Flow Cytometry and TPEF Data Using 3D-PCA

Starting from previously acquired flow cytometry data and TPEF data [23], the phenotypes of the FM models Mac and Mox were compared to those of the reference models. Fifteen parameters were considered: for TPEF data, NADH and FAD AF, Optical Redox Ratio parameter (which represents the [FAD AF]/[NADH AF] ratio) and for flow cytometry data, Positive Cell percentage (PCP) and Mean Fluorescence Intensity (MFI) of the following markers: CD86, CD40, and CD197 (M1 markers), and CD206, CD200R, and CD163 (M2 markers). PCP and MFI were considered first for each marker individually, then in the four defined subsets: CD86^+^/CD206^−^, CD86^+^/CD206^+^, CD86^−^/CD206^+^, and CD86^−^/CD206^−^. MFI, PCP, and AF data were also used as quantitative variables in an original 3D-PCA performed using the FactomineR and pca3d packages in the R software 4.4.1. Experimental conditions served as categorical (qualitative) variable. The mean value for a sample was taken for each variable analyzed in the 3D-PCA. As all markers were not labeled in all flow cytometry acquisitions, missing values (~10% of the whole data) for the corresponding flow cytometry variable were inferred using the missDMA R package before 3D-PCA [48]. This package handles missing values by statistical analysis and then 3D-PCA can be performed with all samples, despite the presence of few missing data. Retaining only the first two dimensions of the PCA was insufficient to adequately capture the variability within the dataset. However, the first three dimensions accounted for approximately 75% of the total inertia and were deemed sufficient to represent the majority of the dataset’s variability. Visualizing three dimensions on a two-dimensional plane typically necessitates the presentation of multiple 2D plots, which individually fail to clearly convey the spatial relationships between different experimental conditions. Consequently, a 3D plot was generated, despite its potential to be somewhat challenging for initial interpretation. To enhance visualization, we provided rotating animated GIFs in Supplementary Figure S1A,B. Moreover, enhanced versions of these figures (Supplementary Figure S2A,B) are included, which further depict the contributions of individual variables to the three principal components. The ellipsoids drawn in the plots represent the dispersion of data points corresponding to each experimental condition, with approximately 80% of the measurements falling within these ellipsoids.

### 4.4. Metabolic Characterization of the Macrophage Experimental Models by Extracellular Flux Analysis

The bioenergetic profiles of the macrophage models were investigated using a Seahorse Bioscience XF96 Extracellular Flux Analyzer (Agilent, Santa Clara, CA, USA) that allows the simultaneous kinetic measurements of Oxygen Consumption Rate (OCR) and ExtraCellular Acidification Rate (ECAR), which reflect respiration and glycolysis, respectively. Cells were plated on 96-well custom plates designed for use with the analyzer (50,000 cells/well) and grown to confluence in complete medium. Then, the different macrophage models were generated as previously described. The day before the assay, the calibration plate was incubated (overnight) with water without CO_2_ for probe hydration. The next day, water was replaced with Seahorse calibration medium (Agilent Seahorse XF-calibrant, Agilent) at 37 °C without CO_2_ for 30 min. Before the experiment, the growth medium was removed, and cells were washed and incubated at 37 °C without CO_2_ in warm enriched Seahorse medium (Seahorse XF medium, 11 mM glucose, 2 mM glutamine, 1 mM pyruvate, 2% BSA with pH adjusted to 7.4) for 1 h. Once the calibration completed, the “Mito Stress” assay was performed by sequential addition of four compounds: 1 μM oligomycin (inhibitor of ATP synthesis), 2 μM carbonyl cyanide 4-(trifluoromethoxy) phenylhydrazone (uncoupling agent), 1 μM rotenone/antimycin A (inhibitors of complex I and complex III of the respiratory chain, respectively). The instrument was set to acquire consecutive measurements for 1 h with mixing in between. Data were analyzed using the Wave Desktop software 2.6.1. The Agilent Seahorse XF Real-time ATP rate assay was performed to measure the rate of ATP production from the two key energy pathways, simultaneously in live macrophages using a label-free technology that delivers a dynamic picture of the cell bioenergetics with indicators of the glycolysis and respiration contribution.

### 4.5. ROS Detection

ROS accumulation was assessed with CellROX^®^ Green (Cat. No.C10444, Thermo Fisher Scientific France) a fluorogenic, ROS-sensitive dye. The reagent measures superoxide (O_2_^−^) and hydroxyl radical (^•^OH) in living cells. This dye becomes fluorescent upon binding to DNA after being oxidized, allowing ROS quantification. CellROX^®^ Green Reagent was added directly to the medium at a final concentration of 5 µM per well and incubated at 37 °C for 30 min. Cells were then fixed with 4% paraformaldehyde solution in PBS for 15 min. After two washes with 1X PBS, 4′,6-diamino-2-phenylindole (DAPI) dihydrochloride (Thermo Fisher Scientific) was used as nuclear counterstain at a concentration of 0.47 µM (Thermo Fisher Scientific). After one wash with 1X PBS 0.1X, the samples were mounted on a slide with Prolong medium (Prolong Diamond Antifade 5, Thermo Fisher Scientific). Excitation and emission were 485 and 520 nm. Fluorescence was measured within 24 h after staining. Images were obtained using a Leica DMI8 inverted camera fluorescence microscope with a 63X/1.4 oil objective at the Bordeaux Imaging Center (BIC). Fluorescence intensity was quantified automatically by the FIJI software 2.14.0.

### 4.6. Ceroid Staining

Ceroids were detected using a hybrid immunochemical/immunofluorescence assay based on GL13, a lipophilic, biotin-linked Sudan Black-B (SBB) analog (SenTraGorTM^®^) (P.Galanis & Co_Lab Supplies, Athens, Greece) [49]. Staining was performed according to the manufacturer’s protocol. Briefly, the reagent was dissolved in 3.75 mL absolute ethanol and incubated in 56 °C water for 120 min. The dissolved dye was kept in the original vial and sealed with parafilm to avoid evaporation of ethanol and stored at room temperature in the dark. Cells were washed twice with 10X PBS, once with 50% ethanol, then once with 70% ethanol at room temperature for 5 min. The SenTraGor^®^ reagent was then filtered using a 0.22 µm filter and added to cells at a concentration of 8.2 mmol/L. After 8 min, cells were washed four times with 50% ethanol, four times with PBS, and then incubated with 0.5%Triton X-100 in 10X PBS for 3 min. This allowed visualizing ceroid staining after removal of other neutral lipids. Then, a rabbit anti-biotin (600–401-098, Rockland Immunochemicals) antibody was used at a concentration of 2.6 μg/mL to directly detect biotin in the SenTraGor^®^ reagent. After incubation with the antibody at 4 °C overnight, cells were washed with 1X PBS three times before incubation with the goat anti-rabbit secondary antibody coupled to Alexa Fluor 488 (ab150077, Abcam, Amsterdam, Netherlands) at a concentration of 2 μg/mL. To show that ceroids are formed in lysosomes, LAMP2 (a lysosome marker) was simultaneously detected using a rat anti-LAMP2 antibody (ab13524, Abcam) at a concentration of 10 μg/mL after saturation in 10% AB serum, 0.1% Tween-20, and 0.1% Triton X-100 at room temperature for 30 min. A goat anti-rat secondary antibody coupled to Cy5 (ab6565, Abcam) was used at a concentration of 2 μg/mL for LAMP2 revelation. After five washes with 1X PBS and 1 wash with 0.1X PBS, Prolong medium (Prolong Diamond Antifade 5, Thermo Fisher Scientific France) containing 2 μg/mL DAPI dihydrochloride (Thermo Fisher Scientific France) was used for mounting the glass coverslips onto slides before imaging with a confocal laser scanning microscope (TCS SP5 multiphoton confocal microscope, Leica Microsystems, Mannheim Germany), with the following wavelengths of excitation and emission, Ex 633 nm/Em 650–750 nm, Ex 488 nm/Em 500–550 nm, and Ex 405/Em 415–460 nm, to detect the fluorescence of LAMP2, ceroids, and DAPI, respectively, in addition to the contrast phase acquisition. Image analysis and fluorescence quantification were performed with the FIJI software 2.14.0)

### 4.7. Polarization Assay

Two protocols of polarization were established: In the first one, Mφ were treated for 48 h with modified LDL and a type-II cytokine cocktail. Alternatively, Mφ were incubated 48 h with modified LDL first, then the cell medium was changed and the cytokine cocktail was added for another 48 h. Following these two polarization processes, phenotypic and functional polarization of Mφ compared to M1 and M2 models and to untreated controls (LDL alone, without adding cytokines) was addressed. In some experiments, α-tocopherol was also added during the incubation with LDL. After stimulation, supernatants were collected and human ELISA kits IL-10 (KE00170-96T), IL-6 (KE00139), IL-18 (KE00193), IL-1β (KE0021) (Proteintech™ Planegg-Martinsried, Germany) and IL-1RA (KAC1181) from Invitrogen, Thermo Fisher Scientific France) were used according to the manufacturer’s instructions. Cells were washed and fixed with 4%PFA, incubated for 20 min in a blocking buffer composed of 1X PBS, 10% FBS, 1% BSA, 0.3M glycine, and 0.1% Tween-20, and stained with an anti-CD40 antibody (rabbit anti-human AB224639, Abcam, and a secondary goat anti-rabbit Alexa 488 antibody: Ex 495/Em 519 nm, AB150077, Abcam), or an anti-CD86 antibody (rabbit anti-human Alexa Fluor^®^ 488: Ex 495/Em 519 nm, ab290990, Abcam), and anti-CD206 antibody (mouse anti-human CD206-Coralite: Ex 593/Em 614 nm, 2A6A10, Thermo Fisher Scientific France). After mounting the coverslips on glass slides in a suitable medium (Prolong Diamond Antifade, Thermo Fisher Scientific) containing 0.47 μM DAPI (Thermo Fisher Scientific France), immunofluorescence images were acquired with the Leica DMI8 (Microsystems, Mannheim Germany), inverted camera system using a 63X/1.4 oil objective at the Bordeaux Imaging Center ((BIC), Bordeaux, France). The TRITC, FITC, and DAPI filters were used. Z-stacks were acquired using 1 μm optical sections. The images were analyzed with FIJI 2.14.0. Brightness and contrast were standardized for all representative images. A program that automates a series of commands on FIJI 2.14.0 (macro) was used for the quantification of fluorescence intensities in the two channels.

### 4.8. Statistical Analysis

Statistical tests and data graphs were performed with the GraphPad Prism 10.0.2 software (GraphPad software). Outliers in each dataset were not considered when data for one experimental condition was missing; mixed-effects models were used to handle the absences and ensure the robustness of the analysis. We primarily applied one-way repeated measures ANOVA for experiments involving a single factor (Figure 4, Figure 5B–E, Figure 7A,D and Figure 11A,B) and two-way ANOVA for experiments involving multiple factors (Figure 1, Figure 5F, Figure 7B,E, Figure 9, Figure 10, Figure 12 and Figure 13). One-way ordinary ANOVA was used for Figure 6. Most ANOVAs were conducted using repeated measures to account for the structure of the data. The resulting histograms represent the mean ± SEM and the *p*-values were considered significant at alpha of 0.05. (NS = *p*-value > 0.05; * *p*-value < 0.05; ** *p*-value < 0.01; *** *p*-value < 0.001 and **** *p*-value < 0.0001).

## 5. Future Directions

We plan to use these models to screen human antibodies for their therapeutic potential: we will compare Mφ status before and after treatment with these antibodies and assess their functional polarization to an immunoregulatory profile. Nevertheless, other therapies focusing on immunoregulation, such those involving cytokines or other M2-promoting agents, can also be considered if properly targeted, thereby minimizing systemic effects while directly modulating Mφ within the plaques. Gene therapy approaches using viral vectors or lipid-based nanoparticles to deliver genes encoding these cytokines to Mφ could also enhance their polarization toward the M2 phenotype. Additionally, drugs already used clinically, such as PPARγ agonists (e.g., pioglitazone), which promote anti-inflammatory gene expression and enhance cholesterol efflux, reducing foam cell formation, could be redesigned or adapted to specifically target Mφ within atherosclerotic plaques. Nanoparticles and liposomes can also be engineered to carry immunomodulatory agents to these macrophages, ensuring targeted and enhanced delivery while minimizing side effects. The administration of these treatments, whether intravenously or locally, could be combined with existing therapies like statins to provide a more comprehensive approach to managing atherosclerosis, potentially reducing plaque progression and cardiovascular risk.

In another study, we are considering exploring nanoparticles loaded with ApoA1 and biomimetics acceptors such as reconstituted HDL to induce cholesterol efflux and using TPEF imaging to monitor changes in the FAD-like AF signal, which would indicate improvements in cholesterol management. In this therapeutic strategy, we plan to provide a thorough characterization of our FM models. This includes understanding the mechanisms driving their lipid uptake and trafficking, such as the levels of free cholesterol and cholesterol esters, the activity of enzymes like ACAT1, and the expression of surface receptors such as ABCA1 (ATP-binding cassette, sub-family A, member 1) and ABCG1 (ATP-binding cassette, sub-family G, member 1). This comprehensive analysis will be essential for comparing the ability of these models to efflux cholesterol in the presence of the acceptors that will be designed in our future studies.

From a diagnostic perspective, TPEF imaging of NADH and FAD-like AF may be valuable for assessing plaque vulnerability. Developing an endoscopic fiber for optical imaging could enable in vivo distinction of pro-atherogenic and anti-atherogenic Mφ to assess coronary plaque vulnerability.

## 6. Limitations of the Study

The aim of experimental FM models is to mimic the FM found in atherosclerotic plaques; however, it is challenging to perfectly replicate all Mφ subpopulations and their interactions. The models characterized in this study represent different positions on the continuum between inflammatory and immunoregulatory Mφ and may represent FM subsets with varying levels of lipid accumulation, as recently identified ex vivo. Despite their limitations, these models are valuable for studying LDL handling dysfunction and screening new therapeutic tools, such as antibodies and/or drugs that can induce immunoregulation or restore cholesterol management and efflux. The characterization of scavenger receptors, cholesterol efflux capacity, and lipid content in these models is necessary for better understanding and future applications.

## 7. Conclusions

Our study demonstrates that cellular NADH and FAD-like autofluorescence (AF) primarily reflect different features of Mφ metabolism and dysfunction. Specifically, NADH AF indicates glycolytic activity, while FAD-like AF correlates with lysosomal ceroid accumulation, a marker of oxidative stress and dysfunctional lipid handling.

The use of 3D-Principal Component Analysis (3D-PCA) has proven to be an innovative approach for distinguishing between different Mφ models based on their functional phenotypes. This technique allows for the effective distinction of Mφ based on their inflammatory versus immunoregulatory characteristics, as well as their autofluorescence profiles associated with energetic and lipid metabolism.

In our models, Mac and Mox FM exhibit distinct phenotypic and functional profiles along the inflammatory-to-immunoregulatory continuum. The Mox model, aligning more closely with inflammatory Mφ, is characterized by elevated FAD-like AF, increased reactive oxygen species (ROS) production, and higher ceroid accumulation. In contrast, the Mac model is more representative of immunoregulatory Mφ, showing a less pronounced inflammatory profile and lower levels of dysfunction.

Both FM models demonstrated potential for polarization toward an immunoregulatory profile through treatment with IL-4 and IL-13. Notably, the inflammatory profile of Mox50 macrophages was significantly attenuated by this cytokine cocktail, with additional improvement observed when α-tocopherol was included, suggesting a potential therapeutic avenue for mitigating Mφ dysfunction.

These findings underscore the relevance of both FM models for studying the mechanisms underlying dysfunctional LDL handling and for developing and testing new therapeutic interventions. Both models are valuable for assessing new drugs and therapeutic strategies designed to reduce inflammation, enhance cholesterol efflux, and restore Mφ function in the context of atherosclerosis. The Mac model may be particularly useful for evaluating treatments aimed at promoting immunoregulation, while the Mox model offers insights into advanced stages of dysfunction and oxidative stress.

## Data Availability

Data is contained within the article or Supplementary Material.

## Supplementary Materials

The following supporting information can be downloaded at: https://www.mdpi.com/article/10.3390/ijms251810146/s1.
