# Supplementary material for: Phenotypic, Metabolic, and Functional Characterization of Experimental Models of Foamy Macrophages: Toward Therapeutic Research in Atherosclerosis
Source: Int J Mol Sci. 2024 Sep 21;25(18):10146. doi: 10.3390/ijms251810146 (PMC11432604; doi:10.3390/ijms251810146)
Supplement: Supplementary file 1 [file ijms-25-10146-s001.zip › Legend of supplemental figures.pdf]

Legend of Supplemental Figures

S1-A: Rotating visualization of Figure 3-A, representing a 3D-PCA analysis incorporating PCP, AF, and MFI data. The data were collected from different donors (n=5 for Mox, n=7 for Mac, and n=13 for M1 and M2).

S1-B: Rotating visualization of Figure 3-B, representing a 3D-PCA analysis based on PCP and AF data. The data were obtained from different donors (n=5 for Mox, n=7 for Mac, and n=13 for M1 and M2).

S2-A: Rotating visualization of Figure 3-A, including the contributions of individual variables to the principal components. This visualization highlights positive and negative correlations between variables, as well as independent variables. It also allows for correlation of experimental conditions with specific variable levels. For example, MFI measurements of CD40 and CD86 show a positive correlation with each other and with the percentage of CD197-positive cells. The data points corresponding to M1-polarized MP align with the direction of these variables, indicating that M1 polarization is associated with high expression levels of these receptors.

S2-B: Rotating visualization of Figure 3-B, showing the contributions of individual variables to the principal components. This representation highlights positive and negative correlations between variables, as well as independent variables. Experimental conditions can be linked to specific variable levels. For instance, MFI measurements of the CD206 and CD200R receptors exhibit a positive correlation with each other and an inverse correlation with autofluorescence in the NADH emission band. This is consistent with the fact that M2 polarization is associated with high expression levels of these receptors and that M2 macrophages rely on OXPHOS rather than glycolysis to generate the energy necessary for their function.
