# Supplementary figures and images for: Phenotypic, Metabolic, and Functional Characterization of Experimental Models of Foamy Macrophages: Toward Therapeutic Research in Atherosclerosis
Source: Int J Mol Sci. 2024 Sep 21;25(18):10146. doi: 10.3390/ijms251810146 (PMC11432604; doi:10.3390/ijms251810146)

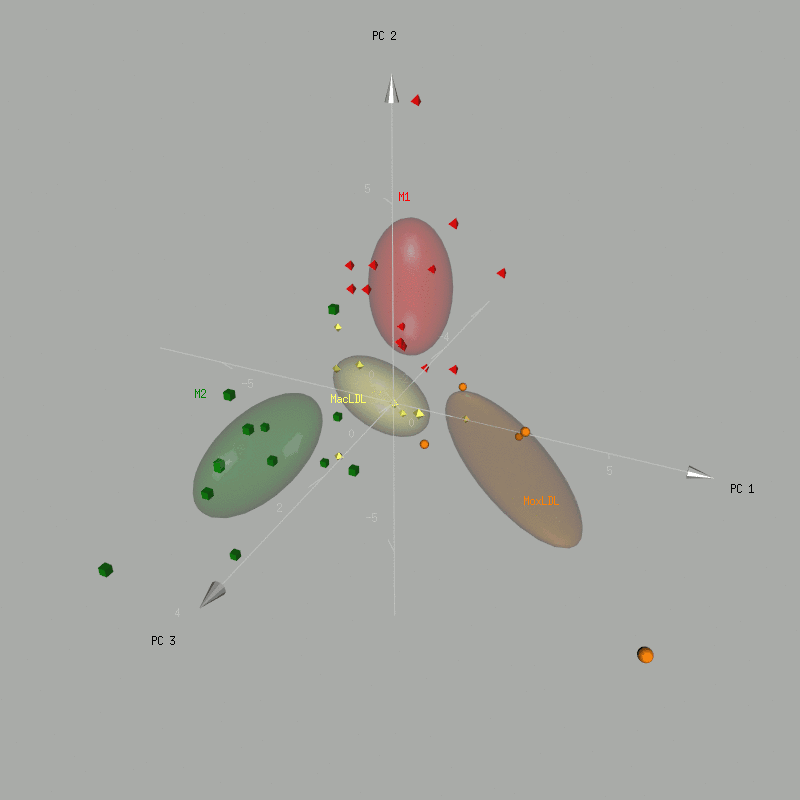

Supplement: Supplementary file 1 [file ijms-25-10146-s001.zip › Supplemental S1-A_Cyto_PCP_MFI_AF_bg_gray_wo_CE.gif]

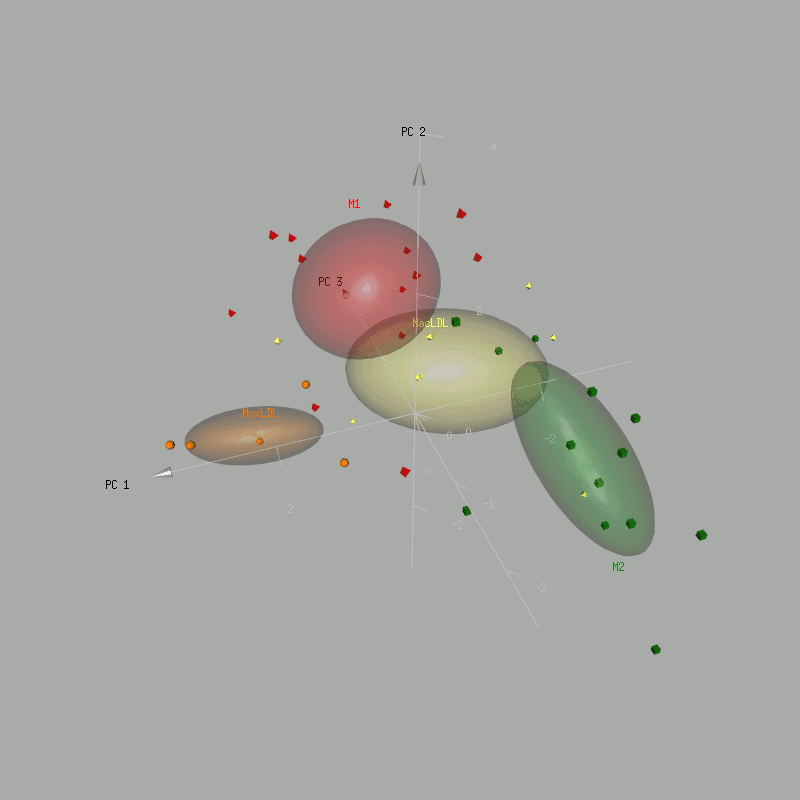

Supplement: Supplementary file 1 [file ijms-25-10146-s001.zip › Supplemental S1-B_Cyto_PCP_AF_bg_gray_wo_CE.gif]

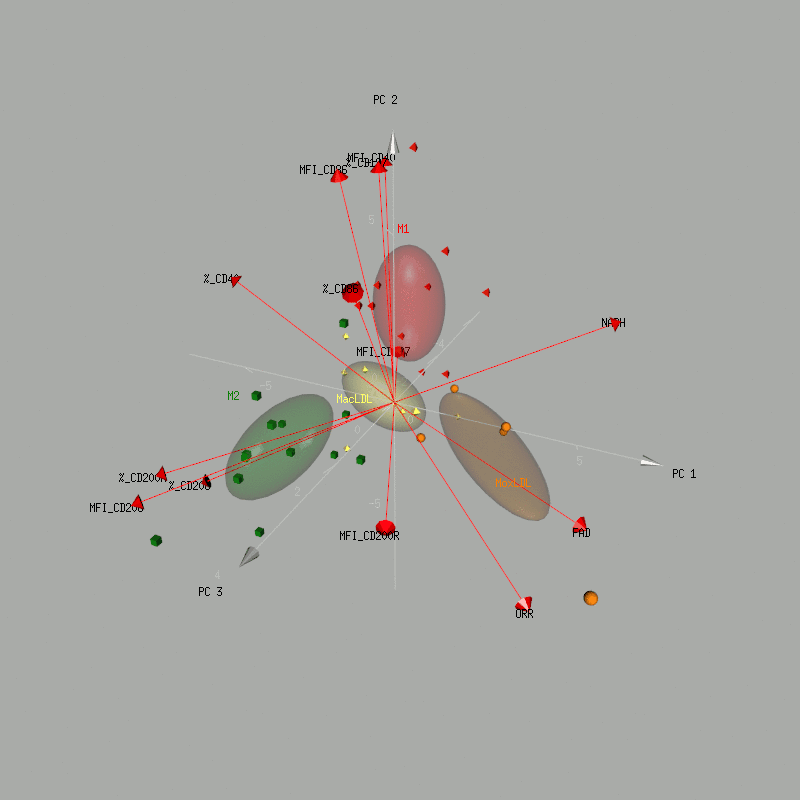

Supplement: Supplementary file 1 [file ijms-25-10146-s001.zip › Supplemental S2-A_Cyto_PCP_MFI_AF_bg_gray_biplots_wo_CE.gif]

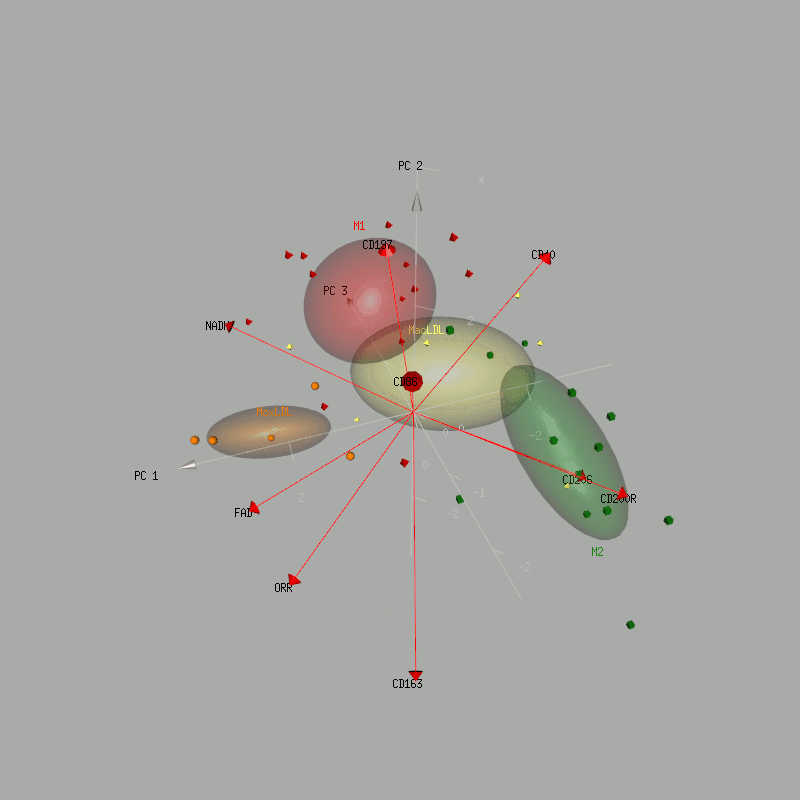

Supplement: Supplementary file 1 [file ijms-25-10146-s001.zip › Supplemental S2-B_Cyto_PCP_AF_bg_gray_biplots_wo_CE.gif]
